# Strengthening tobacco control enforcement in Nigeria: a policy and regulatory analysis supporting an expanded mandate for NAFDAC

**DOI:** 10.1186/s12889-026-27589-6

**Published:** 2026-04-25

**Authors:** Saifuddeen Kamfut Sani, Rametu Momodu, Abbas Bashir Umar, Anees Ya’u Ashiru, Imran Gambo Ibrahim

**Affiliations:** 1 National Agency for Food and Drug Administration and Control, Lagos, Nigeria; 2 Department of Public Health, Ahmadu Bello University, Zaria, Nigeria; 3 Merit Pharmaceuticals Limited, Kano, Nigeria

**Keywords:** Tobacco control, NAFDAC, WHO FCTC, Illicit trade, Regulatory capacity, Nigeria

## Abstract

**Background:**

Nigeria has adopted comprehensive tobacco control legislation, including the National Tobacco Control Act (NTCA) 2015 and Tobacco Control Regulations 2019, yet continues to experience high tobacco-related morbidity and a substantial illicit cigarette market. Institutional fragmentation and weak enforcement capacity, compounded by evolving obligations under the WHO Framework Convention on Tobacco Control (FCTC) and the Protocol to Eliminate Illicit Trade in Tobacco Products, undermine effective implementation.

**Methods:**

A desk-based qualitative policy and regulatory analysis was conducted using three complementary approaches: (1) comparative legal mapping of Nigeria’s tobacco control framework against core WHO FCTC and Illicit Trade Protocol obligations; (2) thematic synthesis of WHO guidance, treaty decisions, and peer-reviewed evidence on institutional arrangements for tobacco regulation; and (3) an implementation-readiness assessment of the National Agency for Food and Drug Administration and Control (NAFDAC) across key capacity domains.

**Results:**

The analysis identified five interrelated structural gaps: fragmented institutional authority; absence of a central product regulator; weak supply-chain and illicit trade control; inadequate protection from tobacco industry interference; and a regulatory vacuum for emerging nicotine products. Nigeria’s current ministry-led configuration constrains compliance with WHO FCTC Articles 9, 10, 11, and 15, while NAFDAC already possesses significant laboratory, port-of-entry, and digital traceability capacity developed through pharmaceutical regulation.

**Conclusion:**

Reconfiguring Nigeria’s tobacco control architecture to assign NAFDAC a central technical regulatory mandate, while retaining health policy stewardship within the Federal Ministry of Health (FMoH), offers a legally feasible and context-appropriate pathway to strengthen enforcement, meet evolving treaty expectations, and reduce tobacco-related harms.

## Introduction

Tobacco use remains a leading preventable cause of morbidity and mortality worldwide, responsible for more than eight million deaths annually, comprising approximately seven million from direct tobacco use and over one million attributable to second-hand smoke exposure, with a growing share of the burden now concentrated in low- and middle-income countries (LMICs) [1–3]. In these settings, formal adoption of comprehensive tobacco control laws often coexists with limited institutional capacity, inadequate enforcement resources, and sustained tobacco industry interference [4]. Nigeria, Africa’s most populous country, ratified the WHO FCTC in 2005 and subsequently enacted the National Tobacco Control Act (NTCA) in 2015 and the Tobacco Control Regulations in 2019, thereby establishing a formal legal framework for smoke-free environments, tobacco advertising, promotion, and sponsorship (TAPS) restrictions, packaging and labelling requirements, and licensing provisions. On paper, this framework aligns closely with core WHO FCTC obligations [5]. In practice, however, persistent gaps between legislative provisions and enforcement outcomes remain evident: illicit tobacco trade is widespread, emerging nicotine products are proliferating with limited oversight, and supply-chain controls remain underdeveloped [6, 7]. A central structural feature of Nigeria’s tobacco control architecture is the fragmentation of regulatory and enforcement authority across multiple agencies. In Nigeria, enforcement agencies involved in tobacco control (e.g., Police, NSCDC, Customs) do not operate under the direct administrative control of the Federal Ministry of Health. Instead, the FMoH exercises coordination and policy oversight functions, while operational enforcement authority remains institutionally dispersed across independent agencies with separate command structures [5]. Ministries of health are generally oriented toward policy formulation and coordination rather than highly specialized technical functions such as product regulation, laboratory-based evaluation, or nationwide market surveillance. In contexts where these technical functions are not institutionally embedded within the ministry or delegated to a clearly empowered agency, this can constrain the country’s ability to fulfil WHO FCTC obligations related to product contents, ingredient disclosures, and illicit trade control [8]. Recent decisions adopted at the Eleventh Session of the Conference of the Parties (COP11) to the WHO FCTC and the Fourth Session of the Meeting of the Parties (MOP4) to the Illicit Trade Protocol have further elevated expectations around operational capacity, including digital track-and-trace systems and oversight of emerging nicotine products. Within this evolving landscape, the National Agency for Food and Drug Administration and Control (NAFDAC) represents a potentially underutilized technical regulator, given its nationwide enforcement presence, laboratory infrastructure, port-of-entry inspection capacity, and experience with product registration and pharmaceutical traceability [9]. This paper addresses a critical gap in the tobacco control literature for LMICs by focusing on institutional design and regulatory capacity rather than solely on legislative content. Specifically, the study pursues three interrelated objectives:

- To map Nigeria’s current tobacco control governance architecture against core WHO FCTC and Illicit Trade Protocol obligations.
- To assess the functional and operational readiness of NAFDAC to assume an expanded mandate for tobacco and nicotine product regulation.
- To develop a context-specific, phased reform pathway that aligns Nigeria’s enforcement system with global regulatory best practices while accounting for political and institutional constraints.

## Conceptual and analytical framework

This analysis is grounded in an Institutional Governance and Regulatory Capacity framework, which posits that effective policy implementation depends not only on the content of the law but also on the design and operational capacity of the institutions tasked with enforcement [10, 11]. Drawing on established concepts in public health governance and regulatory science, the framework emphasizes four interlinked dimensions: legal authority, technical competence, enforcement reach, and insulation from industry interference. Three key analytical pillars underpin this framework. First, institutional fragmentation occurs when regulatory and enforcement responsibilities are dispersed across multiple agencies with overlapping or ambiguous mandates, often resulting in inconsistent enforcement, weak accountability, and duplication of effort [11, 12]. Second, regulatory authority refers to the statutory power vested in an agency to perform functions such as product registration, market authorization, quality control, and facility inspections. In tobacco control, the WHO FCTC and its implementing guidelines require Parties to designate a competent authority for coordination and implementation, but do not prescribe a centralized regulatory structure. However, evidence from implementation studies suggests that where regulatory authority is highly fragmented without a clearly empowered technical lead agency, products may enter the market without systematic pre-market evaluation or consistent oversight [19]. Third, enforcement capacity encompasses the technical and physical resources required to compel compliance, including laboratories, information systems, field offices, port presence, and specialised personnel with investigative and prosecutorial skills. While the WHO FCTC implementing guidelines require a “designated authority” rather than specifying a “central” one, the evidence base consistently demonstrates that dispersed regulatory authority without a clearly empowered lead technical agency leads to systematic enforcement failures [5, 10]. The argument advanced here is not for administrative monopoly but for functional primacy: assigning to one technically competent agency the specific product regulation tasks that require laboratory capacity, registration infrastructure, and supply-chain monitoring expertise, while other agencies retain complementary enforcement roles. The framework suggests that policy effectiveness is maximized when enforcement is centralized or clearly coordinated under a technically competent agency that possesses both the statutory authority to regulate products and the operational capacity for nationwide surveillance and sanctioning [10]. Nigeria’s current configuration, in which the Federal Ministry of Health (FMoH) serves as Competent Authority while NAFDAC retains extensive technical capacity but a limited tobacco-specific mandate, represents a structural misalignment between legal authority and operational capability. This fragmented institutional design creates significant regulatory gaps that may be exploited by tobacco industry actors, ultimately compromising the integrity of national health policies [5, 19, 21]. Importantly, the constraint lies not in the designation of the FMoH as Competent Authority per se, but in the absence of a clearly defined and sufficiently robust technical regulatory mandate for NAFDAC to perform specialised functions such as product evaluation, testing, and supply-chain surveillance.

**Fig. 1:**
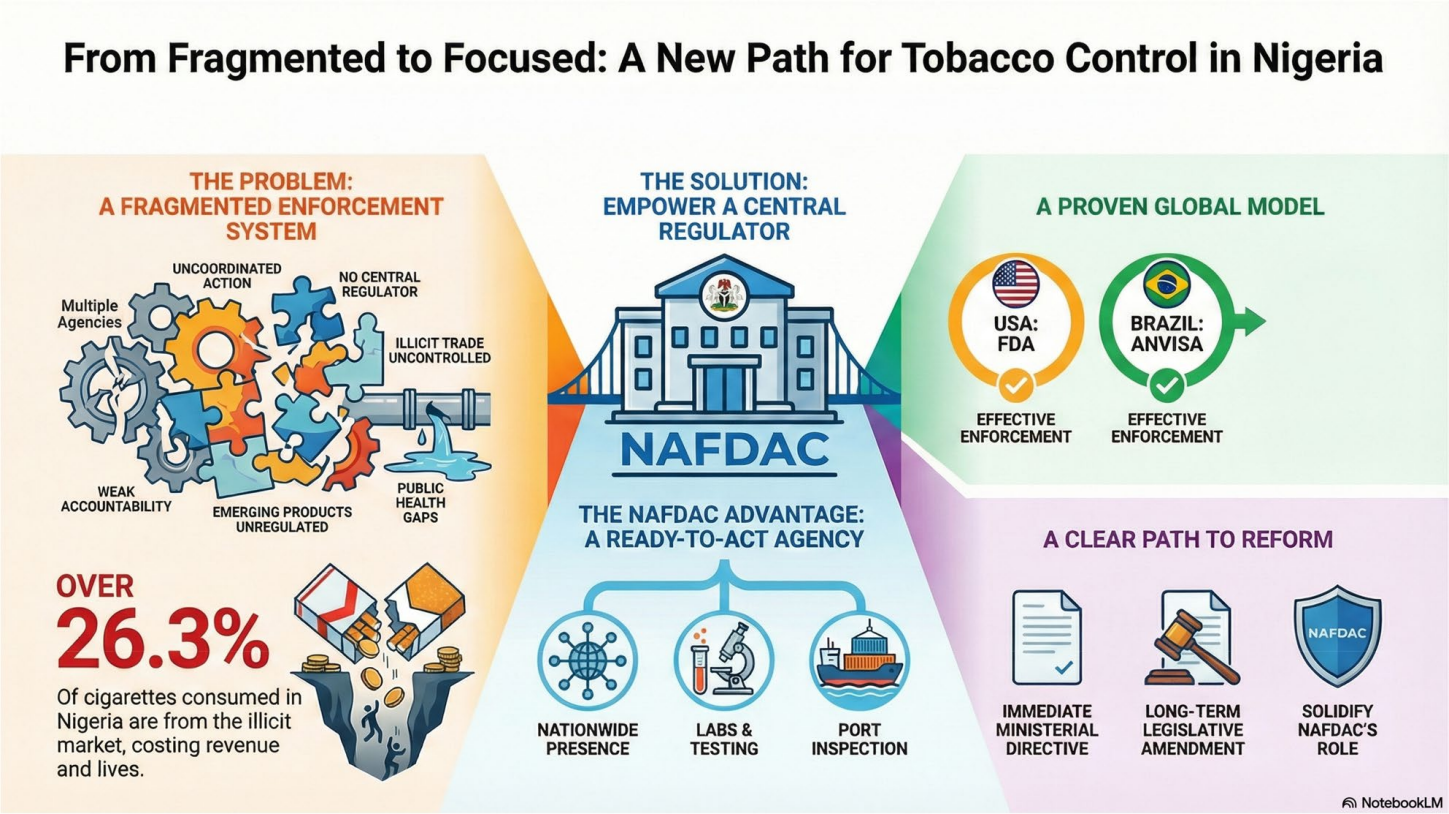
From fragmented enforcement to a NAFDAC-centred regulatory model for tobacco control in Nigeria

Figure 1 illustrates the proposed shift from Nigeria’s current fragmented, ministry-centric tobacco control enforcement structure to a centralized, NAFDAC-centred technical regulatory model, highlighting the alignment of legal authority, technical capacity, and enforcement reach.

### Methods

#### Study design

This study is a desk-based qualitative policy and regulatory analysis that treats laws, regulations, institutional mandates, and treaty obligations as the primary units of analysis rather than individual-level health outcomes. It combines comparative legal mapping, thematic policy synthesis, and an implementation-readiness assessment to examine whether Nigeria’s current governance architecture can realistically deliver on WHO FCTC and Illicit Trade Protocol obligations.

### Data sources and selection

Data were drawn exclusively from secondary sources selected to capture legal, institutional, and normative dimensions of tobacco control. Four main categories of documents were included:

1. Domestic legal and policy instruments:
  • National Tobacco Control Act (NTCA) 2015
  • National Tobacco Control Regulations 2019
  • NAFDAC Act (Cap N1, Laws of the Federation of Nigeria, 2004)
  • Selected national tobacco control policy and enforcement plans where publicly available.

2. International treaties and normative guidance:
  • WHO Framework Convention on Tobacco Control (FCTC) and associated guidelines.
  • Protocol to Eliminate Illicit Trade in Tobacco Products.

It is important to clarify Nigeria’s current status with respect to the Protocol to Eliminate Illicit Trade in Tobacco Products. Nigeria signed the Protocol but has not yet deposited its instrument of ratification and therefore remains a signatory rather than a full Party to the Protocol [17]. This distinction carries significant legal implications: the mandatory enforcement mechanism obligations under Article 15 of the Protocol, including the requirement to establish a national track-and-trace system within a defined timeframe, are legally binding only on States Parties. As a signatory, Nigeria bears a general international law obligation not to act contrary to the Protocol’s object and purpose, and the Protocol’s provisions continue to provide an authoritative normative standard for domestic reform. Ratification of the Protocol should therefore be treated as an immediate policy priority alongside the institutional reforms proposed in this paper.

- Official decisions and outcome documents from COP11 (FCTC) and MOP4 (Illicit Trade Protocol).

3. Institutional and technical documentation:
  • WHO implementation reports and technical guidance on tobacco product regulation, supply-chain control, and Article 5.3.
  • Publicly available documents describing NAFDAC's statutory mandate, organisational structure, laboratory capacity, port-of-entry inspection functions, and digital traceability initiatives (e.g., Mobile Authentication Service and GS1-based pharmaceutical serialisation).
4. Comparative regulatory sources:
  • Peer-reviewed literature and official regulatory documentation describing tobacco product regulation models in the United States (FDA), Brazil (ANVISA), Kenya, and South Africa.

Documents were identified through structured searches of WHO and Nigerian government websites. They targeted searches of PubMed and Google Scholar using combinations of terms such as “Nigeria tobacco control”, “FCTC implementation Nigeria”, “NAFDAC enforcement”, “tobacco track-and-trace”, and “illicit tobacco trade Nigeria”, complemented by snowball sampling from reference lists, consistent with established approaches to legal mapping and health policy implementation reviews [13, 14].

### Analytical approach

The analysis proceeded in three stages.

1. Comparative legal mapping: Provisions related to product regulation, ingredient disclosures, packaging and labelling, and illicit trade were extracted from Nigerian laws and regulations and mapped against WHO FCTC Articles 9, 10, 11, and 15 and relevant Illicit Trade Protocol provisions. The mapping focused on the allocation of regulatory authority, product oversight responsibilities, and enforcement powers across institutions. A clarification on analytical scope is necessary. While Articles 8 (smoke-free environments), 13 (tobacco advertising, promotion and sponsorship), and 14 (cessation) are also central WHO FCTC obligations and are addressed within Nigeria’s legislative framework, this analysis deliberately focuses on Articles 9, 10, 11, and 15 because these require technically specialised regulatory functions, such as chemical testing, ingredient disclosure management, packaging oversight, and supply-chain control, that are most directly aligned with NAFDAC’s existing operational capacities. The intent is not to suggest that Articles 8, 13, and 14 are less important, nor to exclude them from the broader tobacco control agenda, but to analytically isolate the subset of FCTC obligations where gaps in technical regulatory capacity are most likely to arise under a fragmented institutional arrangement [10, 11]. These functions differ fundamentally from population-level public health interventions such as smoke-free enforcement and TAPS restrictions, which remain appropriately within the Federal Ministry of Health’s stewardship and multi-sectoral enforcement framework. Accordingly, the observed gaps related to illicit trade and the proliferation of emerging nicotine products are used as tracer indicators of weaknesses in product regulation and supply-chain control, rather than as a comprehensive assessment of all FCTC implementation domains.
2. Thematic policy synthesis: An iterative thematic synthesis of WHO guidance, treaty decisions, and peer-reviewed literature identified recurring themes affecting enforcement effectiveness, including institutional fragmentation, supply-chain control, protection from tobacco industry interference, and regulation of emerging nicotine products. Nigerian findings were interpreted in light of these themes to identify structural drivers of implementation gaps.
3. Implementation-readiness assessment: An implementation-readiness assessment of NAFDAC was undertaken using a structured matrix of capacity domains: nationwide enforcement presence, port-of-entry inspection capability, laboratory and analytical infrastructure, product registration and market surveillance experience, and digital traceability systems. Each domain was qualitatively rated (limited, moderate, or advanced) based on documentary evidence, with particular attention to parallels between pharmaceutical regulation and potential tobacco control functions. No primary data collection, formal scoring validation, or performance metrics were used. This matrix drew on established readiness and capacity assessment approaches used in health systems, which specify multi-domain frameworks to appraise organizational preparedness for new regulatory functions [15, 16].

A critical analytical caveat is the fundamental difference between pharmaceutical and tobacco regulation. Pharmaceutical regulation operates within a benefit–risk framework, in which authorized products are presumed to offer a net clinical benefit when used as directed, and regulators engage iteratively with manufacturers as part of a legitimate product approval process. Tobacco regulation, by contrast, involves products that are inherently harmful, with no established safe level of consumption. Accordingly, the use of NAFDAC’s pharmaceutical regulatory experience in this analysis is intended solely as an indicator of technical and operational capacity (e.g., laboratory infrastructure, product registration systems, and inspection capability), rather than as a direct analogue of regulatory purpose or industry engagement. The Ministry of Health uniquely retains the authority to recommend or mandate product prohibitions, a prerogative that precedes and may preclude formal NAFDAC jurisdiction, and this policy leadership role must not be diminished. This distinction is acknowledged as a methodological limitation of the present analysis and is discussed further in the Limitations section.

### Methodological limitations

This policy analysis is limited by its exclusive reliance on secondary sources and the absence of primary enforcement data or stakeholder interviews. As a result, it cannot directly quantify enforcement performance or capture informal institutional dynamics such as intra-governmental bargaining and tobacco industry strategies. The implementation-readiness assessment of NAFDAC is based on documented mandates and programs, particularly in pharmaceutical regulation, and does not include new data on human resources, budgetary allocation, or internal organisational culture specific to tobacco control. The findings should therefore be interpreted as a structured institutional and regulatory argument that requires future empirical validation through enforcement metrics, cost-effectiveness analyses, and political economy studies.

## Results

### Overview of Nigeria’s tobacco control governance

Nigeria’s tobacco control framework is built primarily on the NTCA 2015 and Tobacco Control Regulations 2019, which collectively address smoke-free environments, TAPS restrictions, packaging and labelling, and licensing of tobacco-related activities. The NTCA serves as the principal mechanism for domestic implementation of WHO FCTC obligations, while the Regulations operationalise key provisions by specifying licensing procedures, health warning requirements, and enforcement protocols [7].

### Legal framework

The foundation of Nigeria’s tobacco control effort rests on two primary instruments: the National Tobacco Control Act (NTCA) of 2015 and the National Tobacco Control Regulations of 2019 [18]. The 2015 Act is a comprehensive piece of legislation that covers smoke-free environments, the prohibition of tobacco advertising, promotion, and sponsorship (TAPS), and strict requirements for health warnings, licensing, and product disclosure [5]. Crucially, the Act serves as the legal mechanism to give effect to Nigeria’s international obligations under the WHO Framework Convention on Tobacco Control (FCTC). To ensure this Act is functional, the 2019 Regulations operationalise the law by detailing the specific procedures for licensing, the technicalities of health warnings, and the protocols for enforcement [8].

### Institutional architecture

Enforcement responsibility in Nigeria is currently diffused across a complex, multi-agency structure. The Federal Ministry of Health (FMoH) is designated as the Competent Authority, responsible for overall policy and licensing decisions, including packaging and labelling approvals [5]. Policy advice is provided by the National Tobacco Control Committee (NTCC), while operational enforcement is delegated to primary enforcement agencies such as the Police, the Nigeria Security and Civil Defence Corps (NSCDC), and Environmental Health Officers, supported by agencies like the Federal Competition and Consumer Protection Commission (FCCPC), Standards Organisation of Nigeria (SON), Nigeria Customs Service (NCS), and the National Drug Law Enforcement Agency (NDLEA) [8]. Although this multi-agency design promotes multi-sectoral involvement, it creates complex accountability structures and coordination challenges.

## Findings: structural and operational gaps in enforcement

The analysis identified five interrelated gaps that constrain effective implementation.

1. Fragmented institutional authority

Enforcement responsibilities are distributed across numerous agencies with overlapping or ambiguous mandates, and no single authority holds a comprehensive remit from production to retail. This fragmentation leads to reactive, inconsistent enforcement across states and weak accountability, as FMoH relies on agencies that often lack specialised training in tobacco law implementation [5, 19].

2. Absence of a clearly empowered technical product regulator

Under the NTCA, NAFDAC is not assigned primary responsibility for key tobacco product oversight functions such as product registration and market authorization [5]. In practice, this creates a gap in systematic pre-market evaluation and post-market surveillance, as no single technically specialised agency is clearly mandated to receive and assess ingredient disclosures or conduct routine facility inspections [5, 19]. Importantly, this analysis does not suggest that all regulatory authority should be centralised or that the role of the Federal Ministry of Health (FMoH) should be diminished. The FMoH retains the critical policy prerogative to determine whether specific tobacco or nicotine products are permitted, restricted, or prohibited, decisions that logically precede and may preclude the need for technical regulatory evaluation. Rather, the gap identified here relates specifically to the absence of a clearly designated and sufficiently empowered technical authority to regulate products that are legally permitted within the market. In the absence of such technical oversight, products and manufacturers may enter the market without systematic evaluation or consistent regulatory scrutiny, limiting Nigeria’s ability to operationalise WHO FCTC Articles 9 and 10 [5], which require mechanisms for regulating product contents and disclosures.

3. Weak supply-chain and illicit trade control

Nigeria has not yet implemented a national track-and-trace system required under the Illicit Trade Protocol, and licensing regimes for distributors and retailers remain inconsistent. These weaknesses contribute to a substantial illicit cigarette market; estimates suggest that around a quarter of cigarettes consumed in Nigeria in 2016 were illicit, with continued losses in tax revenue and undermining of health policy goals [6].

4. Inadequate protection from industry interference (Article 5.3)

Implementation of WHO FCTC Article 5.3 remains limited in Nigeria, with evidence of unregulated interactions between government actors and the tobacco industry and the continued use of industry-sponsored corporate social responsibility activities. Transparency rules governing such interactions are weak, and conflict-of-interest mechanisms for policymakers remain underdeveloped, increasing the risk that institutional fragmentation may be exploited to delay or dilute enforcement [5, 19, 21]. Evidence of these weaknesses includes the inclusion of the Manufacturers Association of Nigeria (which encompasses tobacco companies) on the National Tobacco Control Committee; the participation of the Standards Organisation of Nigeria (SON)—in which the tobacco industry holds membership, in developing cigarette constituent regulations; the absence of a mandatory public register of tobacco industry contacts with government officials; and the continued allowance of tobacco industry corporate social responsibility activities despite WHO FCTC guidance recommending their prohibition [5, 20]. These findings are corroborated by independent FCTC implementation reviews for Nigeria and broader evidence of industry interference patterns in LMICs [5, 19, 21].

5. Regulatory vacuum for emerging nicotine products

Emerging nicotine products such as electronic nicotine delivery systems (ENDS), heated tobacco products, and nicotine pouches are expanding rapidly in Nigerian urban markets but are not clearly covered by existing regulations. These products often enter the market without chemical or device safety oversight and are marketed online and to youth with minimal restriction, while the current architecture lacks the specialised technical capacity to test e-liquids, devices, or emissions [22, 12].

## Alignment with who fctc obligations

Table 1 summarizes the alignment between key WHO FCTC obligations and Nigeria’s current institutional capacities, highlighting areas where NAFDAC’s existing assets could be leveraged.

**Table 1:** Alignment between WHO FCTC obligations and Nigeria’s institutional capacities

FCTC Obligation (Article)	Functional Requirement	Current Institutional Assignment	Evidence of Gap	Existing NAFDAC Assets
Article 9 – Regulation of contents	Routine chemical testing; product standards; pre-market evaluation	FMoH (policy); no designated technical regulator [5]	No national programme for testing tobacco contents; absence of pre-market registration [7]	Accredited laboratories; experience with pharmaceutical analysis
Article 10 – Regulation of disclosures	Receipt and evaluation of ingredient submissions; secure data systems	FMoH (policy); no evaluation mechanism [19]	No central authority mandated to assess disclosure requirements [23]	Systems for regulated product registration and documentation
Article 11 – Packaging and labelling	Approval of health warnings; border and retail monitoring	FMoH (policy); multiple agencies for enforcement [5]	Limited systematic monitoring of imported and domestically sold products [24]	Port inspection mandate; experience enforcing labelling
Article 15 – Illicit trade & supply-chain control	Licensing, inspections, track-and-trace, data exchange	Multiple agencies (Customs, Police, FMoH) [5]	No national digital track-and-trace system; weak licensing [4]	Mobile Authentication Service; GS1-compliant serialization
Emerging nicotine products	Device and liquid testing; marketing restrictions	No designated authority [22]	Regulatory vacuum and rapid market growth [5]	Laboratory capacity for device and chemical analysis

## International regulatory models and lessons for Nigeria

A review of selected international experiences underscores the importance of assigning clearly defined technical tobacco regulatory functions to specialized agencies. In the United States, the Food and Drug Administration (FDA) exercises comprehensive authority over tobacco products under the 2009 Family Smoking Prevention and Tobacco Control Act, including pre-market authorization, product standards, and enforcement actions, supported by substantial laboratory and field capacity [25, 26]. In Brazil, the National Health Surveillance Agency (ANVISA) plays an analogous role, combining product registration, testing, and market enforcement in a large middle-income setting [27, 28]. Hybrid models in South Africa and Kenya illustrate alternatives in which health ministries retain policy leadership but share or delegate technical functions to specialised agencies, such as revenue authorities or medicines regulators [29, 30]. These experiences suggest that, where ministries lack technical infrastructure, effective implementation depends on formalised collaboration with agencies that possess laboratory capacity, regulatory expertise, and enforcement reach. It is important to acknowledge well-documented criticisms of the FDA tobacco regulatory model. Several analysts have argued that positioning tobacco under an agency whose primary function is product market authorization may inadvertently legitimate a harm-reduction framing over abstention-first public health approaches [31, 32]. The FDA’s Modified Risk Tobacco Product (MRTP) authorization pathway has attracted specific criticism for potentially providing marketing advantages to industry by creating a “reduced risk” category that could undermine cessation efforts [5]. Furthermore, concerns about industry capture, whereby sustained regulatory engagement gradually shapes agency culture toward facilitation rather than restriction, are relevant to any FDA-style model. Any NAFDAC-centred reform for Nigeria must build in explicit institutional safeguards, including strict Article 5.3 compliance, prohibition of industry participation in technical standard-setting processes, and regular independent audits of NAFDAC’s tobacco regulatory decisions, to mitigate these risks [21, 33].

Table 2 summarizes selected international tobacco regulatory models and highlights their institutional configurations, lead technical agencies, and relevance to Nigeria’s tobacco control context.

**Table 2:** Selected international tobacco regulatory models and relevance for Nigeria

Country	Institutional Model	Lead Technical Agency	Key Regulatory Instruments	Relevance for Nigeria
United States	Centralised technical regulator	FDA – Center for Tobacco Products	Pre-market authorisation; product standards; enforcement [25, 34]	Demonstrates feasibility of centralising technical tobacco regulation within a health products agency
Brazil	Centralised technical regulator	ANVISA	Product registration; laboratory testing; marketing restrictions [27]	Offers a middle-income analogue for a NAFDAC-led model
South Africa	Hybrid	Department of Health + SARS	Health regulations + tax and anti-illicit trade measures [29]	Highlights need for structured collaboration between health and revenue authorities
Kenya	Hybrid	Tobacco Control Board + Pharmacy and Poisons Board	Health policy + technical oversight of regulated products [30]	Suggests a model for formal cooperation between FMoH and a technical agency
Nigeria	Fragmented	FMoH as Competent Authority; multiple enforcement agencies	Policy leadership with diffuse enforcement mandates [5, 19]	Indicates limitations of ministry-centric configuration and value of consolidating functions in NAFDAC

## NAFDAC’s implementation readiness

Table 3 presents an implementation-readiness matrix assessing NAFDAC’s current capacity across key regulatory domains relevant to tobacco and nicotine product control. The implementation-readiness assessment indicates that NAFDAC holds several comparative advantages that could be leveraged for tobacco control, albeit with important resource and governance conditions.

**Table 3:** Implementation-readiness matrix for NAFDAC

Capacity Domain	Current Status	Evidence / Examples	Additional Requirements for Tobacco Control
Laboratory and analytical capacity	Advanced	Accredited laboratories; chemical analysis for pharmaceuticals [5, 19]	Development of tobacco-specific methods (including ENDS); staff training; quality assurance aligned with WHO guidance
Port-of-entry and field presence	Moderate to advanced	Port inspections; zonal offices; previous product seizures [24]	Dedicated tobacco inspection teams; integration with Customs risk-profiling and data systems
Nationwide enforcement reach	Moderate	Presence in multiple states; experience with product registration enforcement [8]	Expansion of field staff and inspection coverage to retail outlets and informal markets
Digital traceability	Emerging	Mobile Authentication Service; GS1 serialisation for selected medicines [37, 19]	Adaptation of traceability systems to tobacco products; legal backing for mandatory serialization
Legal enforcement powers	Advanced	Statutory authority to register, seize, and sanction across regulated product categories [20]	Explicit extension of mandate to tobacco and nicotine products via NTCA amendment and subsidiary regulations

Importantly, NAFDAC is not currently a member of the WHO Tobacco Laboratory Network (TobLabNet) [35], which provides standardized, validated methods for testing tobacco product contents and emissions as required under FCTC Articles 9 and 10. Achieving WHO TobLabNet membership or equivalent international accreditation for tobacco-specific analytical methods would be a prerequisite for formal tobacco product testing functions and represents an essential area of investment within the proposed reform pathway [35, 36].

**Fig. 2:**
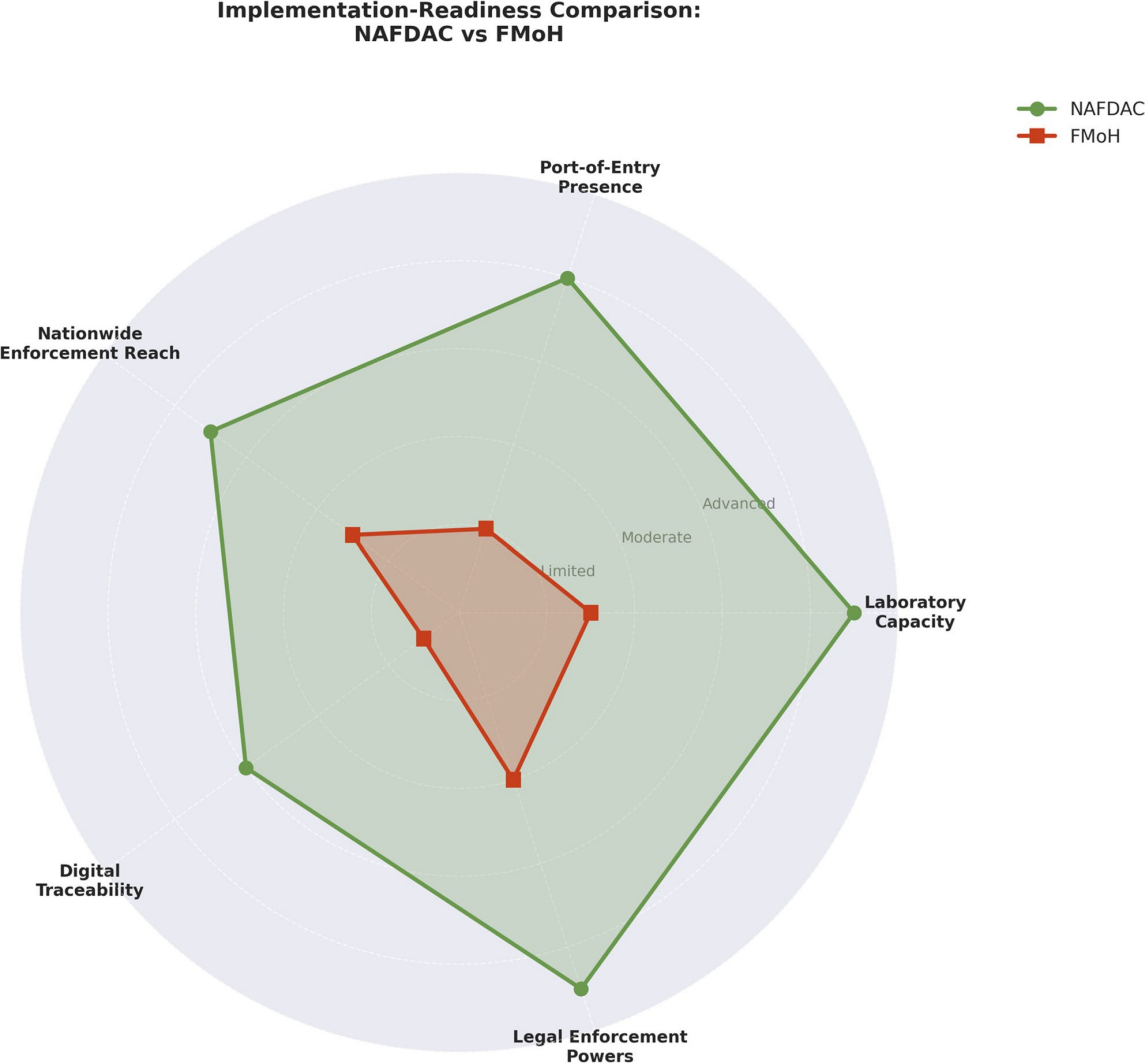
Comparative implementation-readiness of NAFDAC and the Federal Ministry of Health for tobacco and nicotine product regulation in Nigeria

Figure 2 provides a visual comparison of implementation-readiness across technical and enforcement domains, demonstrating NAFDAC’s stronger operational capacity relative to the Federal Ministry of Health.

## Discussion

### Institutional design and enforcement effectiveness

The findings suggest that Nigeria’s principal tobacco control constraint lies in institutional design rather than legislative absence, as robust statutory provisions coexist with limited capacity to perform technically demanding functions such as product regulation and supply-chain control [20, 38]. Designating the FMoH as Competent Authority has created a structural mismatch, since ministries are oriented toward policy stewardship rather than laboratory-based evaluation and nationwide enforcement, and these gaps are unlikely to be resolved through coordination mechanisms alone. Concentrating technical regulatory functions in a specialised agency such as NAFDAC could address several of these gaps by aligning statutory authority with operational capacity, reducing transaction costs in enforcement, and limiting opportunities for industry to exploit institutional fragmentation [20]. At the same time, health policy leadership and strategic oversight would remain within FMoH, preserving its role in setting normative direction, taxation policy, and broader health system integration.

### Implications of evolving treaty obligations

Decisions from COP11 and MOP4 have adopted decisions shifting expectations from formal legal compliance to demonstrable operational capacity. Key COP11 decisions (Geneva, 2025) addressed digital track-and-trace requirements, mandatory reporting on Article 15 implementation, and the regulation of emerging nicotine products; MOP4 advanced progress on licensing frameworks and cross-border data exchange under the Illicit Trade Protocol, particularly in relation to digital track-and-trace systems and oversight of emerging nicotine products [12]. International experience shows that well-designed tobacco track-and-trace systems can reduce illicit trade, improve tax collection, and contribute to lower smoking prevalence [39]. The Illicit Trade Protocol requires a technically competent authority to manage licensing, inspections, and supply-chain monitoring, functions that extend beyond the remit of a policy-focused ministry and draw directly on NAFDAC’s existing strengths in traceability for pharmaceuticals and other regulated products [40, 39]. Failure to adapt institutional arrangements risks leaving Nigeria increasingly misaligned with global standards and foregoing potential health and fiscal benefits from better control of illicit trade and new nicotine products [40]. Conversely, a carefully sequenced expansion of NAFDAC’s mandate could position Nigeria as a regional leader in tobacco regulation.

### Political feasibility and implementation risks

Expanding NAFDAC’s tobacco mandate is politically sensitive and faces several risks. Institutional overburdening is a concern, as NAFDAC already oversees a broad portfolio of food, drugs, and chemicals, and additional responsibilities may dilute core functions without commensurate resources. Political resistance and “turf wars” may arise from agencies that perceive a loss of authority, including the FMoH and enforcement bodies currently involved in tobacco control [29]. Furthermore, capital and recurrent costs associated with laboratory upgrades, staff training, and traceability infrastructure are likely to be substantial. Mitigation strategies include establishing a ring-fenced Tobacco Control Directorate within NAFDAC with dedicated resources, formalising inter-agency coordination through a National Tobacco Enforcement Task Force, and funding implementation through the Tobacco Control Fund and new digital licensing revenues. Importantly, this approach does not seek to replace the existing multi-agency framework, but rather to address fragmentation by assigning clearly defined technical regulatory functions to a specialised agency while strengthening coordination across enforcement bodies. Recognising the risks associated with concentrated regulatory authority, particularly the potential for industry influence, the proposed model incorporates strong WHO FCTC Article 5.3 safeguards. These include mandatory transparency requirements for all interactions with the tobacco industry, strict conflict-of-interest screening for regulatory personnel, and clear limitations on engagement with industry actors. Such measures are essential given that, unlike food and pharmaceutical regulation, tobacco control does not involve products with a legitimate public health benefit and therefore requires a more restrictive approach to regulator–industry interactions [4]. These safeguards are particularly important in contexts where persistent tobacco industry interference often facilitated by weak implementation of WHO FCTC Article 5.3 and institutional fragmentation has been shown to delay or dilute tobacco control policies in many LMICs [41]. A particularly important concern raised in the literature is the structural difference between NAFDAC’s conventional relationship with its regulated constituents in the pharmaceutical, food, and chemical sectors, and the relationship mandated under WHO FCTC Article 5.3 for tobacco. In standard product regulation, iterative engagement between regulators and industry is considered legitimate and necessary for effective oversight. For tobacco, the FCTC explicitly prohibits allowing industry interests to shape public health policy. This means that existing norms of regulatory engagement within NAFDAC would need to be formally and structurally quarantined from the tobacco control function. Failure to do so risks creating precisely the industry capture dynamic that fragmented ministry-led enforcement was also unable to prevent. Operationally, this requires: a dedicated Tobacco Control Directorate that operates under a separate governance protocol; explicit prohibitions on tobacco industry representation in technical advisory bodies; and mandatory disclosure and conflict-of-interest vetting for all NAFDAC officials involved in tobacco regulatory decisions [21, 33].

### Implications for other LMICs

Many LMICs have enacted comprehensive tobacco control laws while retaining fragmented or ministry-centric enforcement arrangements [4]. The Nigerian case underscores the value of systematically assessing whether a technically competent authority exists or can be developed, and of aligning legal mandates, resource allocation, and accountability mechanisms with WHO FCTC obligations. While institutional configurations will vary, the core principle of matching regulatory functions with specialized capacity is widely transferable.

## Recommended reform pathway

### Proposed reform pathway and implementation timeline

A phased reform pathway can help manage political and implementation risks while moving towards a more coherent regulatory architecture. Table 4 outlines a phased reform pathway and implementation timeline for expanding NAFDAC’s technical regulatory mandate for tobacco and nicotine products in Nigeria.

**Table 4:** Proposed reform pathway and implementation timeline

Phase	Timeframe	Action Required	Lead Actor	Expected Outcome
Phase 1	0–6 months	Designate NAFDAC as co-Competent Authority for tobacco and nicotine products under existing NTCA provisions	FMoH	Immediate extension of operational authority for product regulation and enforcement
Phase 2	6–18 months	Develop and roll out regulations for track-and-trace, product registration, and testing protocols for combustible and emerging products	NAFDAC	Improved supply-chain control and product oversight
Phase 3	18–36 months	Amend NTCA to statutorily recognise NAFDAC as primary technical regulator for tobacco and nicotine products	National Assembly	Consolidated and durable central technical mandate
Phase 4	Ongoing	Establish ring-fenced NAFDAC Tobacco Control Directorate; secure sustainable funding; implement Article 5.3 safeguards	Ministry of Finance, FMoH, NAFDAC	Long-term institutional sustainability and protection from industry interference

### Implementation considerations

Implementation should follow a phased rollout, prioritizing the national track-and-trace system and emerging nicotine products by leveraging NAFDAC’s existing traceability expertise. Coordination should be formalised through a National Tobacco Enforcement Task Force, chaired by NAFDAC and including Customs, NDLEA, and the Police. For sustainability, the NAFDAC Tobacco Control Directorate must be funded through the Tobacco Control Fund and new digital licensing revenues. Mandatory transparency for all industry interactions and conflict-of-interest vetting for policymakers are required to protect policy integrity from tobacco industry interference. For Nigeria, aligning technical regulation with NAFDAC represents the most feasible strategy to close the gap between international commitments and national public health outcomes. If only a single reform is politically feasible in the short term, the most impactful step is likely to be the formal designation of NAFDAC as co-Competent Authority for tobacco and nicotine products, coupled with rapid development of technical regulations for product registration and track-and-trace.

### Evaluation and accountability

Given the scale of institutional change proposed, a structured evaluation framework is essential. Formal assessment of implementation progress should be conducted at two years and five years following the commencement of the reform. The two-year evaluation should assess: (i) whether NAFDAC has established functional tobacco-specific laboratory protocols and secured WHO TobLabNet accreditation or equivalent; (ii) the number of tobacco and nicotine products formally registered or assessed; (iii) the operational status of track-and-trace pilot activities; and (iv) the adequacy of inter-agency coordination mechanisms. The five-year evaluation should assess: (i) measurable changes in illicit tobacco market share; (ii) the extent to which NAFDAC’s expanded mandate has been formally legislated through NTCA amendment; (iii) documentation of industry interaction compliance under Article 5.3 safeguards; and (iv) a cost-effectiveness analysis of the transition. Evaluation findings should be submitted to the Federal Executive Council and published to ensure public accountability.

## Conclusion

Nigeria stands at a crossroads where strong tobacco control legislation is undermined by fragmented enforcement arrangements and unclear division of regulatory responsibilities. The analysis indicates that expanding NAFDAC’s mandate to serve as the central technical regulator for tobacco and nicotine products represents a legally feasible and operationally coherent strategy to close existing enforcement gaps. By aligning institutional design with WHO FCTC and Illicit Trade Protocol obligations, Nigeria can strengthen its response to illicit trade, better regulate emerging nicotine products, and enhance protection against tobacco industry interference, with potential benefits for both public health and fiscal policy.

### Limitations

This study is based solely on secondary sources and does not incorporate new empirical data on enforcement performance, human resources, or budgetary allocations, nor does it capture the full complexity of political dynamics surrounding institutional reform. The implementation-readiness assessment of NAFDAC is qualitative and illustrative rather than a formal capacity audit, and recommended reforms may encounter unforeseen political or economic constraints. Nevertheless, the findings provide a structured institutional and regulatory analysis that can inform policy debate, guide administrative reform, and support Nigeria’s efforts to align its tobacco control architecture with evolving WHO FCTC implementation expectations. An additional methodological limitation is the use of NAFDAC’s pharmaceutical regulatory capacity as the primary analogy for tobacco regulation readiness. As discussed above, pharmaceutical and tobacco regulation operate under fundamentally different normative frameworks: the former involves a benefit-risk paradigm with legitimate industry engagement, while the latter requires a principled arms-length relationship with industry as mandated by the WHO FCTC. The readiness assessment is therefore best understood as an evaluation of NAFDAC’s technical infrastructure and organisational experience, not as an endorsement of transferring pharmaceutical regulatory culture wholesale to tobacco oversight. Furthermore, the analysis draws on publicly available documentation of NAFDAC pharmaceutical programmes rather than tobacco-specific operational data, which introduces uncertainty about the transferability of specific capacity estimates.

## Data Availability

The datasets obtained during the current study are available from the corresponding author on reasonable request.

## Abbreviations

ANVISA: Agência Nacional de Vigilância Sanitária (National Health Surveillance Agency, Brazil)
COP: Conference of the Parties
COP11: Eleventh Session of the Conference of the Parties (WHO FCTC)
ENDS: Electronic Nicotine Delivery Systems
FCCPC: Federal Competition and Consumer Protection Commission
FCTC: Framework Convention on Tobacco Control
FDA: Food and Drug Administration (United States)
FMoH: Federal Ministry of Health
GS1: Global Standards One (international supply chain standards organization)
LMIC: Low- and Middle-Income Country
LMICs: Low- and Middle-Income Countries
MAS: Mobile Authentication Service
MOP: Meeting of the Parties
MOP4: Fourth Session of the Meeting of the Parties (Illicit Trade Protocol)
NAFDAC: National Agency for Food and Drug Administration and Control
NCS: Nigeria Customs Service
NDLEA: National Drug Law Enforcement Agency
NSCDC: Nigeria Security and Civil Defence Corps
NTCA: National Tobacco Control Act
NTCC: National Tobacco Control Committee
SARS: South African Revenue Service
SON: Standards Organisation of Nigeria
TAPS: Tobacco Advertising, Promotion and Sponsorship
WHO: World Health Organization
WHO FCTC: World Health Organization Framework Convention on Tobacco Control
